# Posttraumatic Stress Disorder (PTSD) and Instigation of Cardiovascular Events: Ischemic Heart Disease (IHD) and Atrial Fibrillation (AF)

**DOI:** 10.7759/cureus.30583

**Published:** 2022-10-22

**Authors:** Ahmad B Habbal, Chantelle T White, Humaira Shamim, Roba Al Shouli, Lubna Mohammed

**Affiliations:** 1 Cardiology, California Institute of Behavioral Neurosciences & Psychology, Fairfield, USA; 2 Psychology, California Institute of Behavioral Neurosciences & Psychology, Fairfield, USA; 3 Dermatology, California Institute of Behavioral Neurosciences & Psychology, Fairfield, USA; 4 Pediatrics, California Institute of Behavioral Neurosciences & Psychology, Fairfield, USA; 5 Internal Medicine, California Institute of Behavioral Neurosciences & Psychology, Fairfield, USA

**Keywords:** hyper coagulopathy, hypertension, ischemic heart diseases, atrial fibrillation, posttraumatic stress disorder (ptsd)

## Abstract

Posttraumatic stress disorder (PTSD) is a disorder with chronic deterioration that arises after exposure to traumatic events. In these events, a persistent maladaptive reaction was found as a result of severe psychological stress and trauma. It is usually accompanied by mood alteration, disturbing memories, evading behavior, and hyperarousal. Many studies found a connection between PTSD and both ischemic heart disease (IHD) and atrial fibrillation (AF). Impairment of the hypothalamic-pituitary-adrenal axis and sympathetic nervous system can contribute to hypercoagulability, elevated cardiac reactivity, hypertension, dyslipidemia, and chronic inflammation, as all of these processes are implicated in IHD and AF risk. PTSD tends to have a more long‐term course and is associated with more autonomic reactivity rather than a direct negative impact. More research is needed to understand the mechanisms underlying the increased AF risk in patients with PTSD and to identify supposed objectives for screening, intervention, and treatment. Highlighting the connection between PTSD and cardiovascular events would lead clinicians to develop screening tests that might help with the prevention and treatment of cardiovascular events for these patients.

## Introduction and background

Posttraumatic stress disorder (PTSD) is a chronic worsening that is resulted from exposure to traumatic events [1]. This disorder is characterized by persistent maladaptive reactions to severe psychological stress and trauma [2]. It is usually accompanied by mood alteration, intrusive memories, avoidance behavior, and hyperarousal [3]. Traumatic events that may lead to PTSD include violent personal assaults, natural and man-made disasters, and involvement in military combat or warfare [4]. This disorder may cause a malfunction in an individual’s family life, which leads to serious medical, financial, and social problems. To measure PTSD, multiple diagnostic guidelines were developed, including the newest editions of the Diagnostic and Statistical Manual of Mental Disorders (DSM-5) and the International Classification of Diseases (ICD-11). PTSD is mostly diagnosed due to the clinical manifestation of a group of symptoms that appears after exposure to stressors. Its pathogenesis is multifactorial focusing on the activation of the hypothalamic-pituitary-adrenal (HPA) axis factor as an indirect contributory factor to ischemic heart disease (IHD) and atrial fibrillation (AF) [5]. Studies concluded that acute and chronic PTSD patients show an increase in basal heart rate and blood pressure. The increase in heart rate and blood pressure was mostly in response to stimuli that remind them of the trauma. Stimuli vary from loud sounds to visual cues [6].

PTSD patients in both veteran and nonveteran populations are at increased risk of hypertension, hyperlipidemia, obesity, and cardiovascular disease (CVD) [6]. Moreover, the increased activity of the sympathoadrenal axis through the effects of catecholamines on the heart, vasculature, and platelet function could contribute to CVD. There is a reported link between PTSD, diabetes, and hypertension, plus other cardiovascular risk factors, which may establish the linkage between PTSD and heart disease such as AF [6]. IHD has been defined as a new onset of coronary artery disease, angina, or myocardial infarction- by ICD-9 and ICD-10 diagnostic codes [7]. Chronic stress syndromes such as PTSD have known risk factors for AF [3].

AF is considered the most common cardiac arrhythmia, affecting more than 33 million adults worldwide. The growth of this public health issue is a financial burden for both patients and families. Moreover; it is associated with substantial morbidity, mortality, and healthcare cost. As a result, priority should be placed to identify and control the modifiable risk factors for AF. Risk factors such as age, hypertension, diabetes mellitus, obstructive sleep apnea, and lifestyle factors can be measured by using the CHA₂DS₂-Vasc (congestive heart failure, hypertension, age ≥75 (doubled), diabetes, stroke (doubled), vascular disease, age 65 to 74, and sex category (female)) score [3]. 

Recent evidence points to the fact that psychological stress and negative emotions, such as acute anger and hostility, are linked to the initiation and development of AF [3]. Biological data from animal studies provide evidence for this potential link. Biological data also indicate that acute social stress can instigate sympathetic arousal and initiate atrial arrhythmias [3].

**Figure 1:**
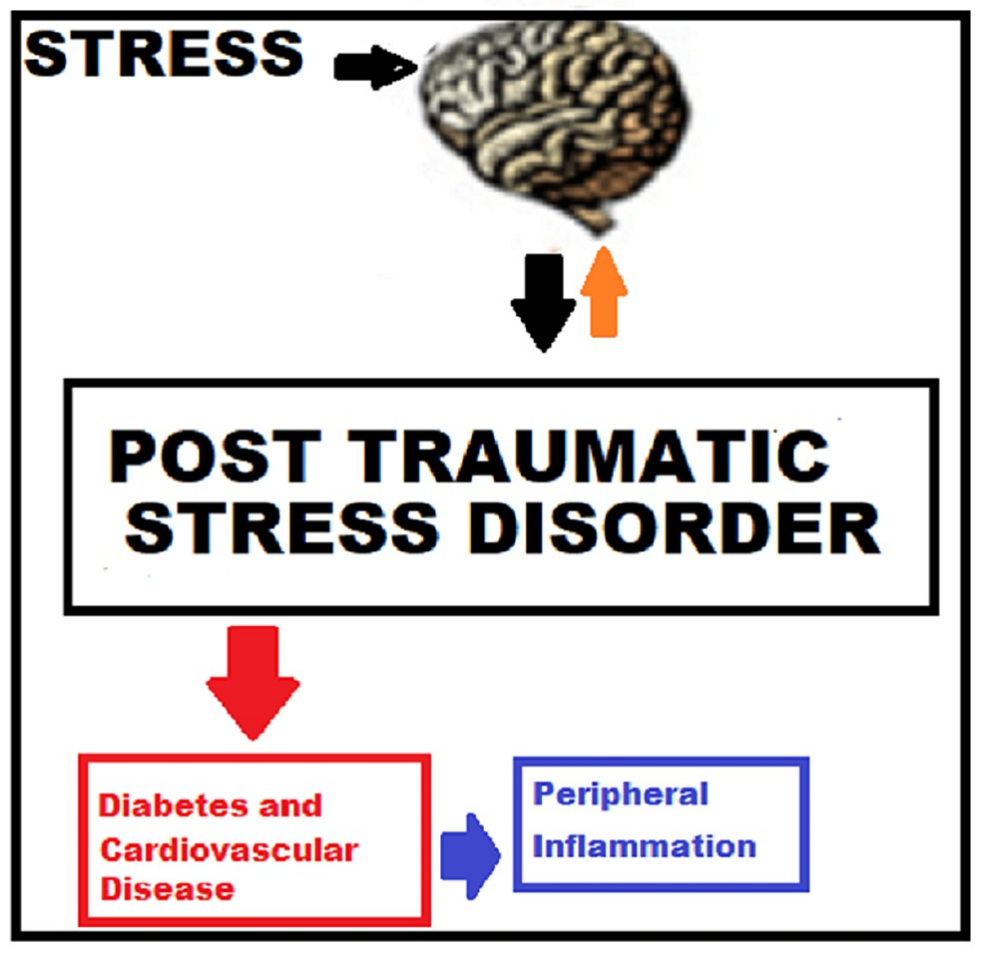
Linkage between PTSD and cardiovascular disease; peripheral inflammation as indirect outcome of PTSD PTSD: posttraumatic stress disorder Image credit: Ahmad Habbal

Understanding both direct and indirect linkage between PTSD and cardiovascular events leads clinicians to develop screening tests that might help with the prevention and treatment of cardiovascular events in advance for these patients. The scope of this review lies in understanding the pathway from PTSD to CVD as in Figure 1

## Review

PTSD is known through a cluster of signs and symptoms that is manifested clinically in patients due to exposure to life-threatening traumas, reexperiencing symptoms (e.g., nightmares, flashbacks, intrusive memories), avoidance symptoms (e.g., trauma reminders, amnesia to details of events), negative cognitions and mood (e.g., emotional detachment, negative worldview, decreased interest in activities), hyperarousal symptoms (e.g. sleep disturbance, hypervigilance, easy startle, irritability), and duration of greater than one month. Multiple guidelines were recently developed to measure PTSD as those in the DSM-5 and ICD-11. Kadiyala, in a review article, lists mnemonics for diagnostic criteria of DSM-5 mental disorders [8].

Diagnosis for PTSD was first given in the DSM-3 published by the American Psychiatric Association in 1980, which has proven to be effective in the research. The 1987 and 2000 DSMs have been improved to the most recent version, DSM-5 (American Psychiatric Association, 2013). As an alternative, the 11th revision of the WHO's ICD-11 is a complete diagnostical tool. ICD adopts a public health perspective, organizes it, and maximizes its use clinically worldwide [9].

Risk factors that are contributory to PTSD include military combat, sexual trauma, conflict and displacement, physical activity, medical illness (e.g., myocardial infarction (MI), stroke, ICU stay), and childhood abuse. Women are twice as likely as men to develop PTSD, with a lifetime prevalence of 10-13% among women in the general population and 12-22% among veteran women [2].

PTSD and alternation in the cardiovascular system

Studies show evidence of connections between PTSD and major risk factors for CVD, such as hypertension and diabetes, as well as major CVD outcomes, such as MI and heart failure. However, there is no clear evidence that these associations are causal or confounded [10].

A prospective study showed that a diagnosis of PTSD was associated with a hazard ratio (HR)= 1.12 (95%CI 1.08-1.17, p < 0.0001) for hypertension diagnosis alone in the electronic medical record, an HR = 1.30 (95%CI 1.26-1.34, p < 0.0001) for a hypertension diagnosis and/or prescription for antihypertensive medication, and an HR = 1.27 (95%CI 1.25-1.30, p < 0.0001) for these occurrences and/or blood pressure in the hypertensive range on two back-to-back medical visits in approximately of 200,000 United States military veterans of the Iraq and Afghanistan conflicts [11].

In an event of stressful stimuli, PTSD patients show increased heart rate and blood pressure. It is also reported in these patients a change in autonomic and HPA axis regulation, which causes glucocorticoid receptors to become more sensitive to negative feedback, and therefore, responsiveness to glucocorticoid decreases [12].

PTSD and hypertension are cross-sectional and linked to a diagnosis of hypertension, which is a significant risk factor for CVD, AF, and stroke. A prospective study showed a 38% increase in the odds of hypertension diagnosis by a primary care provider among the recent veterans of Afghanistan and Iraq for >4.5-year median follow-up [13]. A cohort study showed a 33% increase in the odds of self-reported hypertension at the three-year follow-up among 55,000 active duty and reserve/national guard members in the United States with multiple combat exposures and PTSD was not scaled [13]. A study shows a twofold increase in the prevalence of hypertension among those with PTSD compared to those without. Similar results were found in a registry of >300,000 veterans of wars in Afghanistan and Iraq recently [14].

**Figure 2:**
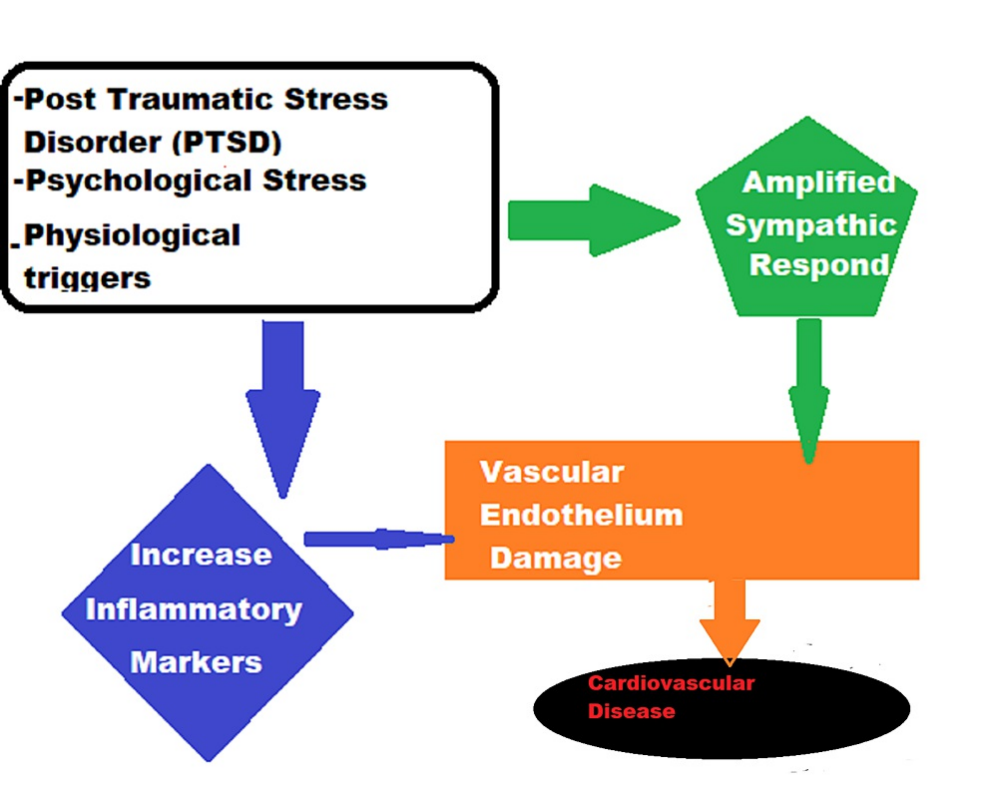
Psychological stress results in amplified sympathetic response (green color), such as higher concentrations of circulating catecholamines, decreased cardiac vagal control, and baroreflex impairment. PTSD inflammatory markers, such as TNFα and interleukin 1β and platelet reactivity to physiological triggers. All pathways that are involved in vascular regulation and damage lead to CVD risk. PTSD: posttraumatic stress disorder; CVD: cardiovascular disease Image credit: Ahmad Habbal

Researchers found that autonomic impairment is proven by the amplified sympathetic response to psychological stress, higher concentrations of circulating catecholamines, decreased cardiac vagal control, and baroreflex impairment [11]. Researchers found a dose-response relationship between PTSD symptom severity and levels of circulating inflammatory markers, such as TNFα and interleukin 1β, in addition to the amplification of platelet reactivity to physiological triggers [11]. All pathways that are involved in vascular regulation can contribute to CVD risk. Both direct and indirect mechanism(s) by which chronic and acute stress, as part of PTSD, contribute to CVD risk have contained a focus on the vascular endothelium [11]. Endothelium reacts to circulating and hemodynamic factors through the release of bioactive substances affecting the vascular tone. The endothelial lack of appropriate response to hemodynamic and circulating factors, as in the earliest stages of CVD, would provide an independent index to a high CVD risk before the clinical manifestation. According to the study, endothelial impairment during or post periods of emotional stress contributes to PTSD and causes alternation in the cardiovascular system CVS [6]. As part of endothelial dysfunction, norepinephrine causes vasoconstriction and function synergistically with endothelin-1 (ET1) as hinted in emotionally triggered cardiac events. ET1 is the most endogenous vasoconstricting protein as it mobilizes from plaque-resident macrophages. Exaggeration of noradrenergic responses during daily stress and trauma reminders in PTSD patients serve as a risk factor for CVD and cardiovascular events through vasoconstriction [11]. Therefore, the increase in the activity of the sympathoadrenal axis contributes to CVD through the effects of catecholamines on the heart, vasculature, and platelet function. Moreover, the elevated levels of circulating catecholamines alter platelet function, through their action on alpha-2a receptors on platelet membranes, causing an increase in platelet aggregation besides other changes in platelet function. As result, it is concluded that there is a link between chronic stress, increased sympathoadrenal activation, and CVD [13]. Figure 2 illustrates this process.

Studies in clinical and in animal models proved the effects of traumatic exposures or chronic stress on the HPA axis. The results of these studies showed that PTSD can cause important neurobiological and psychophysiological changes. Physiological dysregulation of the HPA axis contributes to a higher chance of cardiovascular risk factors in persons with PTSD [13].

Clinical studies found that PTSD has an increased effect on lipid metabolism. Karlovac et al. examined total cholesterol, low-density lipoprotein (LDL), high-density lipoprotein (HDL) cholesterol, and triglycerides among PTSD patients of Croatian war veterans. Those veterans with PTSD had higher levels of cholesterol, LDL cholesterol, and triglycerides, on average, and lower HDL cholesterol levels as compared with other psychiatric patients. The study observed elevated levels of total cholesterol and triglycerides among police officers with posttraumatic stress disorder in Brazil [11].

These findings of cardiologic significance may develop over time as a result of hypertension, hyperlipidemia, and events such as the rupture of atherosclerotic plaques and thrombus formation [13].

PTSD-inducing IHD

In a cohort study of >17,000 adults, Dong et al. concluded that individuals with childhood exposure to a high number of traumatic events such as abuse and neglect were at > 3.5-fold increased risk for ischemic heart disease, impartial from other risk factors such as smoking, poor diet, and sedentary lifestyle [11].

To establish the linkage between PTSD and IHD, a cohort study was performed on 398,769 women from a veteran population. The study concluded the following: (i) PTSD is a risk factor for IHD after ruling out any other IHD risk factors such as obesity, chronic kidney disease, neuroendocrine disorders, and other mental health disorders; (ii) High IHD risk related to PTSD is most noticeable among younger women < 40 years old; and (iii) the risk was stronger among ethnic and racial minority women (Table 1) [14].

**Table 1:** Stratified Analyses of PTSD and Incidence of IHD Based on Age, Race, and Ethnicity for the Sample Size, n = 398,769 Source: Ebrahimi et al., 2021, JAMA Cardiology [15]; Reprinted with permission from the American Medical Association PTSD: posttraumatic stress disorder; IHD: ischemic heart disease

Women Veteran Population		
Subgroup Analysis	Without Posttraumatic Stress (n=265,846)	With Posttraumatic Stress (n=132,923)
Age-stratified		
Age at index visit <40 y		
No. of women	141 128	67 224
No. of IHD events	697	626
Cox proportional hazards survival model, HR (95% CI)	1 (Reference)	1.72 (1.55-1.93)
Age at index visit 40-49 y		
No. of women	63,093	36,599
No. of IHD events	1800	1750
Cox proportional hazards survival model, HR (95% CI)	1 (Reference)	1.58 (1.48-1.69)
Age at index visit 50-59 y		
No. of women	44,216	23,214
No. of IHD events	1940	1501
Cox proportional hazards survival model, HR (95% CI)	1 (Reference)	1.38 (1.29-1.49)
Age at index visit > or = 60 y		
No. of women	17,409	5886
No. of IHD events	1122	504
Cox proportional hazards survival model, HR (95% CI)	1 (Reference)	1.24 (1.12-1.38)
Race-stratified		
White		
No. of women	152,239	77,511
No. of IHD events	3673	2892
Cox proportional hazards survival model, HR (95% CI)	1 (Reference)	1.35 (1.29-1.42)
Black		
No. of women	76,893	42,038
No. of IHD events	1475	1173
Cox proportional hazards survival model, HR (95% CI)	1 (Reference)	1.49 (1.38-1.62)
Other		
No. of women	10,979	6140
No. of IHD events	150	159
Cox proportional hazards survival model, HR (95% CI)	1 (Reference)	1.66 (1.33-2.08)
Ethnicity-stratified		
Hispanic/Latina		
No. of women	19,642	10,837
No. of IHD events	194	175
Cox proportional hazards survival model, HR (95% CI)	1 (Reference)	1.50 (1.22-1.84)
Non-Hispanic/Latina		
No. of women	224,967	116,592
No. of IHD events	5096	4017
Cox proportional hazards survival model, HR (95% CI)	1 (Reference)	1.38 (1.35-1.46)

The same study analysed further to understand the linkage between PTSD and unfavorable cardiovascular findings and concluded that PTSD-diagnosed women veterans have a 44% higher rate of developing IHD in comparison to those without PTSD. Table 2 shows the HR for PTSD with IHD for sample size, n=398,769 [15].

**Table 2:** Hazard Ratio (HR) for PTSD with IHD for the sample size, n = 398,769 PTSD: posttraumatic stress disorder; IHD: ischemic heart disease Source: Ebrahimi et al., 2021, JAMA Cardiology [15]; Reprinted with permission from the American Medical Association

Female Veteran Population			
Variable	Full analytic Sample (n=398,769)	Without Posttraumatic Stress Disorder (n=265,846)	Without Posttraumatic Stress Disorder (n=132,923)
No. of IHD events (person-years)	9940(2,448,660)	5559(1,587,990)	4381(860,670)
Crude Incidence per 1000 person-years	4.06	3.50	5.09
Time to IHD, mean (SD), ya	6.1 (4.5)	6.0 (4.4)	6.1 (4.5)
Age at IHD, mean (SD), ya	56.8 (10.3)	57.8 (10.7)	55.5 (9.7)
Cox proportional hazards survival model, HR (95% CI)	NA	1[Reference]	1.44 (1.38-1.50)

**Figure 3:**
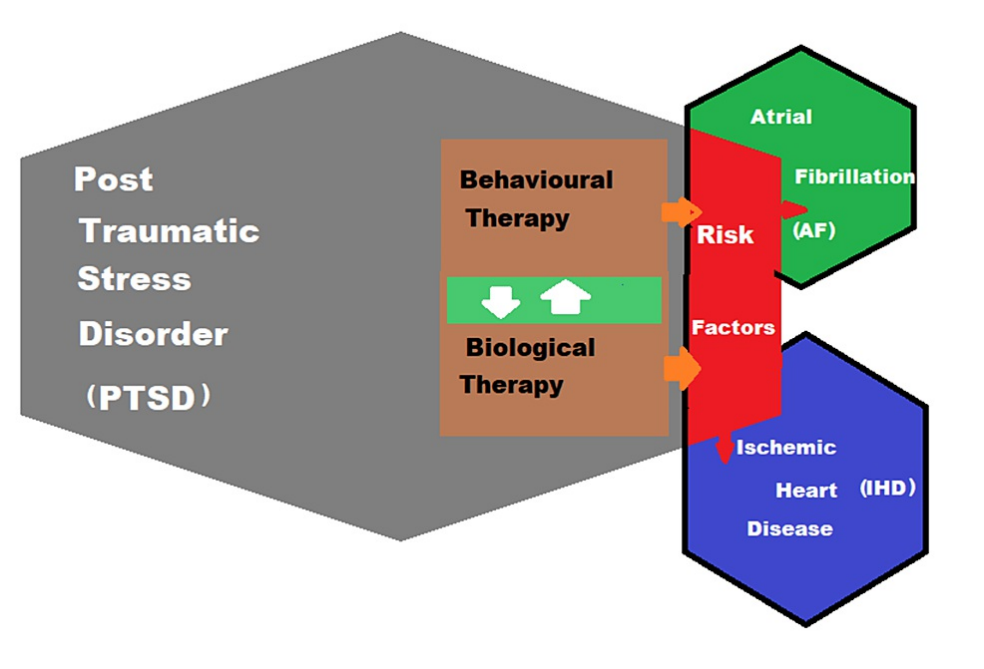
PTSD interactions with different interrelated pathways (brown color) that influence various risk factors (red color) such as hypertension, smoking, sedentary lifestyle, poor diet, insomnia and obesity. PTSD: posttraumatic stress disorder Image credit: Ahmad B Habbal

Investigating biological and behavioral pathways helps in explaining how PTSD could lead to IHD. PTSD is interrelated to the behavioral risk factors and settings for IHD, comprising smoking, sedentary lifestyle, poor diet, insomnia, and obesity [16-18]. Figure 3 shows PTSD interactions with different interrelated pathways that are influenced by various risk factors [11]. 

Moreover, impairment of the sympathetic-adrenal medullary system and HPA axis have been noted in PTSD patients [19,20]. These disturbances could cause harmful effects on metabolic, immune, and cardiovascular systems. For example, impairment of the HPA axis and sympathetic nervous system can contribute to an increase in coagulation, cardiac reactivity, hypertension, dyslipidemia, chronic inflammation, and all processes involved in IHD risk [21]. Genetic factors have a vital role in the PTSD-IHD linkage [22]. In addition, PTSD often exists with other psychiatric conditions (e.g., depression), which are associated with greater IHD risk [23,24]. If PTSD triggers these psychiatric conditions, they could mediate associations between PTSD and IHD onset and progression. Comprehensive tests for these behavioral and biological mechanisms in future studies will help clarify key fundamental methods and identify potential goals for validation and intervention.

PTSD-inducing AF

AF is a non-synchronizing atrial activation with subsequent unsuccessful contractions. AF takes different forms in terms of duration and patterns of termination. AF is considered paroxysmal when terminated a week from onset, whereas it is considered persistent when it last longer, and “long-standing persistent” AF last more than 12 months [25].

By 2030, AF cases in the United States are expected to exceed 12 million. As the aging population grows in number, AF cases upsurge. Approximately 51.2% of cases in the European Union in 2016 were seen in individuals 80 years or older. Counting on the higher proportion of “silent” and thus undetected cases, the number of AF cases would be even greater [25].

The mechanisms by which PTSD increases susceptibility for AF consist of a combination of behavioral/lifestyle and other pathophysiologic factors. PTSD may indirectly prompt individuals to develop AF through the onset or development of hypertension, diabetes mellitus, inflammation, and/or metabolic syndrome. Lifestyle factors and unhealthy behaviors, such as smoking, alcohol consumption, sedentary lifestyle, poor diet, and drug abuse, are found in PTSD patients and may contribute to AF. Other studies suggest that PTSD may trigger the incidence of AF directly through increasing sympathetic activation and decreasing vagal stimulation, which can alter atrial electrophysiological characteristics by shortening the effective refractory period and thereby facilitating AF. Findings from studies have shown that acute negative emotions can precipitate AF [3]. PTSD tends to have a longer duration course and is correlated with greater autonomic responsiveness than transient negative affect. Understanding the mechanisms underlying the elevation of AF risk in PTSD patients should be the aim of future research by seeking the best outcome from screening, intervention, and treatment [3].

Limitations

Our study is characterized by limitations to English language research papers and date of publication, not before 2006. This study is a secondary review using PubMed, Google Scholar, and JAMA research databases. Only free full articles were reviewed disregarding any animal studies. Few studies have investigated the direct linkage between PTSD and AF as well as PTSD and IHD. Most research done in this field is limited to a population from certain demographics, traumatic events, and/or geographic areas. As PTSD patients could be also among displaced populations from war and/ or natural disasters, further studies are needed for these. No studies were found that investigate the differences in the linkage between PTSD and AF with that of PTSD and IHD.

## Conclusions

The risk for an early AF and IHD increases as a result of PTSD. Studying risk factors, that is contributory to AF and IHD in PTSD patients is considered an important clue and linkage to the field of cardiology. Given that heart disease may develop over time as a result of hypertension, hyperlipidemia, and events such as the rupture of atherosclerotic plaques and thrombus formation. Both behavioral and biological mechanisms require a thorough empirical test in any upcoming studies to clarify vital primary methods and reveal future goals for treatment and intervention. Further research is needed to understand the mechanisms behind the increase in AF and IHD risk in PTSD patients. In addition to, establishing possible objectives for screening, intervention, and treatment. Knowledge of the direct and indirect linkage between PTSD and cardiovascular events helps clinicians develop screening tests that might help with the intervention and treatment of cardiovascular events as earlier as possible for these patients. Suggestions for future research include finding the cause-effect relationship between PTSD and IHD along with PTSD and AF with a larger sample size population. looking into risk factors independently and how each of these risk factors contributes to or cofound with PTSD, AF, and IHD. Future studies on PTSD must include individuals from different backgrounds, races, demographic, and types of traumas.

## References

[1] Beristianos MH, Yaffe K, Cohen B, Byers AL (2016). PTSD and risk of incident cardiovascular disease in aging veterans. Am J Geriatr Psychiatry.

[2] Han JK, Yano EM, Watson KE, Ebrahimi R (2019). Cardiovascular care in women veterans. Circulation.

[3] Rosman L, Lampert R, Ramsey CM (2019). Posttraumatic stress disorder and risk for early incident atrial fibrillation: a prospective cohort study of 1.1 million young adults. J Am Heart Assoc.

[4] O'Donnell CJ, Schwartz Longacre L, Cohen BE (2021). Posttraumatic stress disorder and cardiovascular disease: state of the science, knowledge gaps, and research opportunities. JAMA Cardiol.

[5] Bremner JD, Moazzami K, Wittbrodt MT (2020). Diet, stress and mental health. Nutrients.

[6] Coughlin SS (2011). Post-traumatic stress disorder and cardiovascular disease. Open Cardiovasc Med J.

[7] (2022). PTSD Independently Linked to Ischemic Heart Disease. https://www.medscape.com/viewarticle/814375.

[8] Kadiyala PK (2020). Mnemonics for diagnostic criteria of DSM V mental disorders: a scoping review. Gen Psychiatr.

[9] Brewin CR, Cloitre M, Hyland P (2017). A review of current evidence regarding the ICD-11 proposals for diagnosing PTSD and complex PTSD. Clin Psychol Rev.

[10] Miao XR, Chen QB, Wei K, Tao KM, Lu ZJ (2018). Posttraumatic stress disorder: from diagnosis to prevention. Mil Med Res.

[11] Burg MM, Soufer R (2016). Post-traumatic stress disorder and cardiovascular disease. Curr Cardiol Rep.

[12] Dorobantu M, Onciul S, Tautu OF, Cenko E (2016). Hypertension and ischemic heart disease in women. Curr Pharm Des.

[13] Granado NS, Smith TC, Swanson GM (2009). Newly reported hypertension after military combat deployment in a large population-based study. Hypertension.

[14] Cohen BE, Marmar C, Ren L, Bertenthal D, Seal KH (2009). Association of cardiovascular risk factors with mental health diagnoses in Iraq and Afghanistan war veterans using VA health care. JAMA.

[15] Ebrahimi R, Lynch KE, Beckham JC (2021). Association of posttraumatic stress disorder and incident ischemic heart disease in women veterans. JAMA Cardiol.

[16] Remch M, Laskaris Z, Flory J, Mora-McLaughlin C, Morabia A (2018). Post-traumatic stress disorder and cardiovascular diseases: a cohort study of men and women involved in cleaning the debris of the world trade center complex. Circ Cardiovasc Qual Outcomes.

[17] van den Berk-Clark C, Secrest S, Walls J, Hallberg E, Lustman PJ, Schneider FD, Scherrer JF (2018). Association between posttraumatic stress disorder and lack of exercise, poor diet, obesity, and co-occurring smoking: a systematic review and meta-analysis. Health Psychol.

[18] McLay RN, Klam WP, Volkert SL (2010). Insomnia is the most commonly reported symptom and predicts other symptoms of post-traumatic stress disorder in U.S. service members returning from military deployments. Mil Med.

[19] Yehuda R (2006). Advances in understanding neuroendocrine alterations in PTSD and their therapeutic implications. Ann N Y Acad Sci.

[20] Pitman RK, Rasmusson AM, Koenen KC (2012). Biological studies of post-traumatic stress disorder. Nat Rev Neurosci.

[21] Wentworth BA, Stein MB, Redwine LS (2013). Post-traumatic stress disorder: a fast track to premature cardiovascular disease?. Cardiol Rev.

[22] Sumner JA, Duncan LE, Wolf EJ (2017). Letter to the editor: posttraumatic stress disorder has genetic overlap with cardiometabolic traits. Psychol Med.

[23] Cohen BE, Edmondson D, Kronish IM (2015). State of the art review: depression, stress, anxiety, and cardiovascular disease. Am J Hypertens.

[24] (393-406). Drug Abuse and Addiction in Medical Illness: Causes, Consequences and Treatment. Spring.

[25] Ladwig KH, Goette A, Atasoy S, Johar H (2020). Psychological aspects of atrial fibrillation: a systematic narrative review : Impact on incidence, cognition, prognosis, and symptom perception. Curr Cardiol Rep.

